# Respectful Maternity Care during Labour and Postpartum in a Tertiary Hospital: A Descriptive Cross-sectional Study

**DOI:** 10.31729/jnma.8610

**Published:** 2024-06-30

**Authors:** Heera KC, Surya B. Parajuli, Alina Gurung, Anjali Mishra

**Affiliations:** 1 School of Nursing, Birat Medical College Teaching Hospital, Tankisinuwari, Morang, Nepal; 2 Department of Community Medicine, Birat Medical College Teaching Hospital, Tankisinuwari, Morang, Nepal; 3 Hemodialysis Unit, Birat Medical College Teaching Hospital, Tankisinuwari, Morang, Nepal

**Keywords:** *labour*, *maternity care*, *postpartum*, *prevalence*, *tertiary hospital*

## Abstract

**Introduction:**

Respectful maternity care (RMC) is a fundamental human right for women globally. Providing respectful care during labor and postpartum is crucial for the health and well-being of both mothers and newborns. The interactions between healthcare providers and women play a significant role in shaping their future healthcare decisions. Therefore, this research aims to evaluate the prevalence of RMC during labor and postpartum from the patient's perspective at a tertiary care center.

**Methods:**

We conducted a descriptive cross-sectional study at a tertiary center from February 20, 2023, to September 30, 2023. Ethical approval was obtained from the Institutional Review Committee of the same institution. A total of 217 patients were included using consecutive sampling techniques. Data were collected through interviews using a structured questionnaire on respectful maternity care. The point estimate was calculated with a 95% Confidence Interval.

**Results:**

The prevalence of overall respectful maternity care (RMC) score was 81%. The score for right to confidentiality and privacy during labor was 91.7%, treated with dignity and respect was 90.87%, received equitable care free of discrimination was 86.41%, protection from physical harm and ill treatment was 84.02%, while protection of right to information/informed consent and choice'preference was 72.55%.

**Conclusions:**

This study demonstrated a high prevalence of respectful maternity care, with most patients experiencing protection of confidentiality, dignity, equitable treatment, safety, and informed consent, indicating effective implementation of RMC practices at our tertiary care center.

## INTRODUCTION

Respectful Maternity Care (RMC) is a fundamental human right ensuring dignified treatment for women during childbirth.^1^ Despite its importance, global statistics show 41.6% of women experience abuse or discrimination in maternity care.^2^

In Nepal's Kaski district, 83% of women lacked RMC.^3^ Nepal aims to increase institutional delivery to 90% by 2030, up from the current 79%. Achieving this goal requires enhanced maternal care, which can be facilitated by implementing RMC.^4^

This type of study is crucial for obtaining baseline data and enhancing quality of services and implementation of safe motherhood programs. The valuable evidence obtained from this study supports our center for decision-making in areas such as training health professionals, improving institutional setup, and strengthening RMC. Recognizing the importance of this, we aimed to study prevalence of RMC during labor and postpartum period among postnatal women in a tertiary hospital in eastern Nepal.

## METHODS

We conducted a hospital based descriptive cross sectional study at the Department of Obstetric and Gynecology, Birat Medial College and Teaching Hospital, from February 20, 2023, to September 30, 2023. Ethical approval was obtained from the Institutional Review Committee of the same Institute (IRC-PA-279/2023). Participants were informed about the objective of the research and voluntary informed consent was obtained before data collection. Postnatal women admitted in the active phase of labour and delivered at the obstetric department of Birat Medical College Teaching Hospital were included. Women with a history of spontaneous vaginal delivery (SVD) or vacuum delivery with or without tear or episiotomy and without neonatal complications were included for the study. Women delivered by caesarean section, delivered outside the institute (home/vehicles/referred from other hospitals) having comorbid illness and obstetric complications requiring specialised care, critically ill newborns and those unwilling to provide consent for the study were excluded. Sample size was calculated based on the study conducted in Gandaki Medical College, Kaski which reported 17% of participants received RMC.^3^

We enrolled 217 participants after calculating sample size based on Cochrane formula n= Z^2^pq/e^2^, at 95% level of significance and allowable error (l) at 5%.

The tabulated value of Z at 95% level of significance is 1.96, Z^2^ = (1.96)2 = 3.84; prevalence(p) = 17%^3^, q = 100-p = 83; e^2^ = 25. The calculated sample size was 217. Participants were enrolled using a conveniencesampling technique where consecutive samples were taken until the required sample size was met.

Data was collected using a validated tool developed as part of a respectful maternity care toolkit by Maternal and Child Health Integrated Program(MCHIP).^5^ The tool was initially developed in English version, we translated it in Nepali and Maithili language and back into English to check the consistency of the translated tool.^6,7^ The translated tool was pretested in 10% of the samples to ensure validity. Chronbach's alpha was calculated to measure the reliability of the tool, which was 0.801. The pretested tool was then used for data collection by interview technique. The total time required for each participant was approximately 20 minutes.

The questionnaires consisted of seven categories:

1. Protection from physical harm or ill treatment,
2. Protection of right to information, informed consent and choice/preference
3. Protection of confidentiality and anonymity
4. Treated with dignity and respect
5. Received equal, equitable and discrimination free care
6. Never left without care or detained and
7. Non detention or confinement against woman's will.

The questionnaires within each category were developed by reviewing similar literature and standard guidelines on RMC.^1^ Component 1 consisted of 12 items, similarly components 2 to 7 included 16, 1, 5, 2, 4 and 1 items respectively. Total items were 41. Women were considered to have received RMC if they responded yes to each question of the RMC components. Participants having positive responses to the RMC related questions were ranked 1 and those having negative responses were ranked 0. The prevalence of receiving RMC in each component, was achieved as (sum of the score in each component) × 100/ (maximum attainable total score in each component) × (total number of study participants). The total score was calculated by summing each component obtained by each participant and it ranged from 16-41. The prevalence of respectful maternity care was calculated as: (sum of the score of each participant) × 100 / (maximum attainable total score) × (total number of study participants). Greater the percentage of the RMC, the greater was the RMC perceived by participants.^5^

Collected data was entered in Microsoft excel sheet for analysis. Frequency, mean percentage, prevalence and point estimation at 95% confidence interval was calculated.

## RESULTS

A total of 1302 patients had vaginal delivery during our study period. Among them 217 (16.66%) patients were included for the study. The overall prevalence of overall respectful maternity care score was 81% (. Out of 217 deliveries, 124 (57.14%) women were in the 21-25 years age group, 14(6.45%) were aged below 21 years and 22 (10.12%) were illiterate. Among 217 patients, 138 (63.59%) of women were primiparous and four patients had vacuum delivery (Table 1).

**Table 1:** Sociodemographic data of the patients (n= 217).

Variables	n (%)
**Age in years**	
<20	14 (6.45)
21-25	124 (57.14)
26-30	59 (27.19)
31-35	17 (7.83)
35-40	3 (1.38)
**Education**	
Formal	187 (86.17)
Informal	8 (3.69)
Illiterate	22 (10.12)
**Parity**	
Primiparous	138 (63.59)
Multiparous	79 (36.41)
**Mode of delivery**	
SVD	213 (98.16)
Vacuum Delivery	4 (1.84)

From components of RMC, the score for protection of confidentiality and privacy during labour was 91.71%, treated with dignity and respect was 90.87%(Table 2).

**Table 2:** Calculated values for different components of Respectful Maternity Care during labour and postpartum.

RMC components	%
Protection from physical harm or ill treatment	84.02
Protection of right to information, informed consent and choice/preference	72.55
Protection of confidentiality and privacy during labour	91.71
Treated with dignity and respect	90.87
Received equitable care and free of discrimination	86.41
Never left without care	89.86
Never detained or confined against her will	100

## DISCUSSION

Our study revealed an 81% prevalence of Respectful Maternity Care (RMC) during labour and postpartum, surpassing previous findings in Nepal, which ranged from 17% to 84.70%^3,7,8,9^ The higher prevalence underscores a positive trend and encourages the pursuit of enhanced quality services. This is further supported by better equipped and staffed hospitals with adequate facilities, private rooms and medical supplies. This also suggests us to progress towards achieving a higher degree of quality services. Conversely, a systematic review in India reported a low RMC practice of 28.7%, highlighting the need for improvement.^10^ A hospital based study and a systematic review showed very low prevalence of RMC in Ethiopia which was (35.8%) and(48.44%) respectively.^6,11^

Notably, our setting exhibited comparatively better RMC practices, emphasising commitment to providing equitable, non-discriminatory, and compassionate care to every individual patient. We extensively examined the prevalence of Respectful Maternity Care (RMC) across seven distinct categories.^1,12^ Our analysis spanned from the active phase of labour admission through childbirth to the postpartum period, distinguishing our study from others in Nepal that typically focus on labour and childbirth components.^3,9^ Notably, 84.02% of women reported protection from physical harm or ill-treatment in our study, while findings from Nobel Medical College indicated even higher levels at 97.1%.^8^ Conversely, studies in Gandaki Medical College and Kathmandu reported lower percentages of abuse-free care (69.3% and 53%, respectively).^3,9^ A systematic review from India highlighted verbal and physical abuse, threats, and discrimination during labour and childbirth.^10^ In Ethiopia, a hospital-based study reported 76.5% of women being protected from physical harm, contrasting with our higher prevalence.^6^ Our study also revealed a significant proportion (72.55%) of patients perceiving their right to information, informed consent, and choice/preferences, contrasting with lower percentages in studies conducted in Pokhara (32%).^7^ Furthermore, a substantial majority in our study reported protection of confidentiality and privacy during labour (91.7%) and received dignified and respectful care (90.87%), in contrast to a Pokhara study with lower prevalence (77.5%).^7^ In contrast to our findings, a comparative study in Biratnagar revealed a significant prevalence of disrespect and abuse, with all participants reporting non-consented care, 72% experiencing nondignified care, and 66.6% receiving non-confidential care.^13^ Our study, however, showed that 90% of patients never felt abandoned or neglected, a higher percentage than a Pokhara study (74%).^7^ All women in our study experienced non-detention, aligning with previous studies in Pokhara and Biratnagar.^7,13^ While the majority (86.41%) received equitable and non-discriminatory care in our study, a notable 13.41% perceived discriminatory care. In comparison, non-discriminatory care was reported by the majority in Pokhara, Nepal Medical College, and Biratnagar (98.7%, 80.7%, and 67.7%, respectively).^7,9,13^ Another study in Nobel Medical College Teaching Hospital in Biratnagar reported 100% non-discriminatory care.^8^ An EN-Birth study from Pokhara highlighted increased practices of respectful maternity care, ensuring care without abuse, stigma, or discrimination, effective communication, and non-refusal of care.^14^

The comprehensive comparison across and within countries underscores the incomplete delivery of respectful maternity care, revealing gaps in quality maternity care for pregnant and postpartum women. The increasing prevalence in each component of RMC emphasises the importance of protecting the human and reproductive rights of every woman. Analysing the quality of services delivered by healthcare institutions is crucial, with variations in Respectful Maternity Care (RMC) often stemming from factors such as healthcare provider attitudes, provider-to-patient ratios, workload, management policies, and RMC awareness among staff.^15^ Disrespect and abuse during childbirth are global concerns, transcending income levels and locations.^15^ Comparisons with an Ethiopian study highlight differences in all components of RMC prevalence with our study. The possible cause may be attributed to variations in awareness among healthcare professionals, supply and demand side barriers such as inadequate space and infrastructures challenges, inadequate and incompetent staffing, cultural ethnic diversity and socioeconomic status which needed to be further studied.^6,15^ The underreporting of disrespect in our study may result from a lack of awareness about its meaning, given that RMC encompasses a broader range of components for both mothers and newborns.^16^ Disrespectful behaviour from health care providers discourages pregnant women from seeking healthcare, erodes trust and is associated with adverse maternal and neonatal outcomes.^17^ Promoting institutional delivery through RMC is pivotal for enhancing timely care-seeking behaviour and ensuring maternal and neonatal well-being.

Our study is limited by solely relying on patients' perspectives, potentially leading to under or overrated responses and information bias. Cultural and social practices influencing women's understanding of disrespect could have been explored more thoroughly through qualitative research methods, representing a study limitation. The single-centre focus may limit the generalizability of our findings to the entire population. To enhance generalizability, we recommend conducting similar studies at multiple centres with a larger and more diverse population. A mixed-methods approach would provide a deeper understanding of women's perceptions of respectful and disrespectful maternity care. Additionally, future research should consider the healthcare provider's perspective and explore factors hindering the delivery of respectful maternity care.

## CONCLUSIONS

This study demonstrated a high prevalence of respectful maternity care, with most patients experiencing protection of confidentiality, dignity, equitable treatment, safety, and informed consent, indicating effective implementation of RMC practices at our tertiary care center.
